# Antibacterial activity of *Enterococcus faecium* derived from Koopeh cheese against *Listeria monocytogenes *in probiotic ultra-filtrated cheese

**Published:** 2014

**Authors:** Hassan Hassanzadazar, Ali Ehsani, Karim Mardani

**Affiliations:** 1 *Deputy of Food and Drug, Urmia University of Medical Sciences, Urmia, Iran; *; 2 *Department of Food Hygiene and Quality Control, Faculty of Veterinary Medicine, Urmia University, Urmia, Iran.*

**Keywords:** *Enterococcus faecium*, *Listeria monocytogenes*, Probiotic, Ultra-filtrated cheese

## Abstract

Viability of probiotic bacteria in food during maintenance and time of consuming in food has become a challenge in food hygiene and technology and is important for representing their beneficial health effects. The aim of this study was to determine the survival of probiotic *Enterococcus faecium *derived from Koopeh cheese added to industrial Iranian ultra-filtrated (UF) cheese and screening for antimicrobial activity of *Enterococcus faecium* against *Listeria monocytogenes* during two months of cheese ripening. Physiochemical and standard microbial methods were used for isolation of *Enterococcus *strains in cheese samples. The initial number of lactic acid bacteria (LAB) as starter culture was 6 Log g^-1^ in control samples. The counts started to decrease slightly after day seven (*p < *0.05) and dropped to 5 Log g^-1^ at the end of 60 days. The count of LAB in the test groups decreased to 11 Log g^-1^ on the day 60 of ripening. The number of *Enterococcus faecium *was 6 Log g^-1 ^on the day 60. The count of *Listeria monocytogenes *after 60 days of ripening in blank sample decreased 1 Log but in test samples with protective strain decreased 3 Log in 30 days and reached to zero at 45 days. There were not significant (*p <* 0.05) changes in chemical parameters such as fat, protein and total solid of UF cheese treatment groups. The results showed that *Enterococcus faecium* of Koopeh cheese was suitable for development of an acceptable probiotic UF cheese and could be adapted to industrial production of UF cheese.

## Introduction

Chemical preservatives application in food formulation has made consumers’ demand for more natural and minimally processed food. Identifying of specific probiotic strains provides useful quality control tool for food manufactures.^1^^,^^2^ Nowadays, lactic acid bacteria (LAB), as starter cultures, were used to improve both quality and safety of fermented foods.^3^ The enriched foods with probiotics has become a challenge in food hygiene and technology because of survival and viability of this group of bacteria during maintenance and time of consuming.^4^^,^^5^ Survival of probiotic bacteria in foods for exerting their beneficial effects is important.

High pH, fat content, buffering capacity and the solid matrix in cheeses makes them as a suitable carrier than other fermented dairy products for probiotic bacteria. This features can provide good condition for bacteria survival during maintenance of the foods and bacterial passage through the human body.^5^^,^^6^

Various probiotic bacteria mainly lactobacillia and bifidobacteria groups and recently enterococci genera are used in functional foods.^7^^-^^9^ Enterococci as ubiquitous microbes inhabiting soil, food, water and gastrointestinal tract of humans and animals. Genus *Enterococcus *currently comprises more than 26 species. *Enterococcus faecalis*, *E. faecium *and *E. durans *are predominant species most frequently found in dairy products.^10^ Their important role in cheese making and contribution to the promotion of the sensory characteristics is due to proteolytic and lipolytic activity, citrate utilization and aromatic volatile compounds production in various traditional cheeses in the Mediterranean countries.^11^ They are also present in other fermented food products, such as sausages and olives.^11^ From the technological point of view, they play an important role in the production of various traditional cheeses in Iran, Europe and other parts of the world, such as: Lighvan, water-buffalo Mozzarella, Feta, Venaco, Cebreiro and Cheddar cheeses.^3^^,^^12^^-^^14^

The use of probiotics or their antimicrobial compounds in foods are new approach for controlling of food borne pathogens such as *L. monocytogenes.*^15^^,^^16^ Because of inhibitory effects of enterococci strains on *Listeria and Staphylococcus* genera, they would be potential candidate for protective culture. They could be considered as additional biopreservative hurdles for the listeria growth inhibition in fermented foods and be of practical use in food industry.^11^ There are many studies on food enrichments with probiotics and their antibacterial effects on *L. monocytogenes* and other pathogens particularly in fermented dairy products such as Beyaz cheese, Gouda cheese, Cottage cheese, Argentinean cheese, cheddar cheese, whey and ice-cream or frozen yogurts.^4^^,^^15^^,^^17^^-^^26^

Koopeh cheese as a semi-soft type cheese made mainly from raw ewes’ milk and less commonly of cows’ milk or their mixture without starter culture addition in clay jugs is one of the traditional fermented dairy products in some areas of Iran with predominant population of enterococci.^27^

Iranian ultra-filtrated (UF) white cheese as a feta cheese type is the most important type of cheeses with high consumption in Iran. Iranian UF white cheese classified as semi hard cheese with using rennet and starter culture in cheese making protocol. Limited studies are available about the enrichment of the UF cheeses with native probiotics or other useful bacteria isolated from different natural sources particularly fermented foods, such as traditional fermented dairy products in Iran.^28^

The aim of this study was to determine the survival of probiotic *E. faecium* identified at species level by RFLP- PCR derived from Koopeh cheese in Iranian UF white cheese witch produced industrial during two months of cheese ripening and screening period for antimicrobial activity of *E. faecium* against *L. monocytogenes*.

## Materials and Methods

**Fig. 1:**
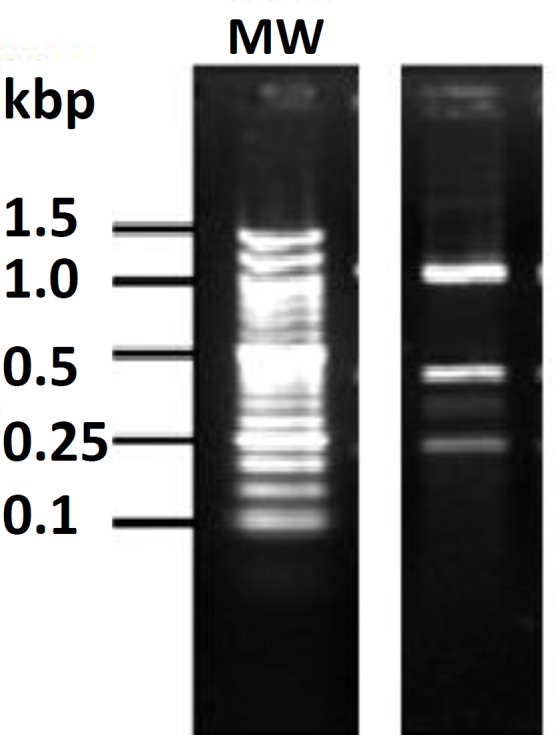
Profiles of *Enterococcus faecium *strain. Amplicons were obtained with universal primers (Scarpellini *et al.*) that digested with restriction enzyme *Hinf І.*^33^

**Isolation of** ***Enterococcus faecium. ***The strains used in this study were a part of LAB that previously isolated from Koopeh cheese samples.^27^ LAB isolated by homogenizing of 10 g cheese with 90 mL 2.0 % (w/v) sodium citrate solution (pH = 7.5, 42 ˚C) in a blender (Stomacher 400; Seward, London, UK) for 2 min. Serial dilutions were made in 0.1 % peptone water solution and plated on specific media for isolation. Lactobacilli were isolated on MRS agar (deMan, Rogosa and Sharpe agar; Merck, Darmstadt, Germany), lactococci were isolated on M17 agar (Merck, Darmstadt, Germany), Isolation of *Enterococcus *strains in cheese samples was done by standard microbiology methods using selective medium of kanamycin esculin azide agar (Merck, Darmstadt, Germany) and then identified by physicochemical methods.^12^^, ^^29^^,^^30^ For gram-positive and catalase-negative isolates further identification was performed by using the following physiological tests: the growth at different temperatures 15 ˚C and 45 ˚C in MRS broth (*Bacilli* and *Coccobacilli*) or 10 ˚C and 45 ˚C in M17 (*Cocci*) broth for 3 days; the growth in M17 or MRS broth with 4.0 % and 6.5 % (w/v) NaCl for 3 days; a gas production from glucose was determined in MRS broth containing inverted Durham tubes; hydrolysis of Arginine by Nessler’s reagent (Merck, Darmstadt, Germany) and fermentation of carbohydrates including arabinose, raffinose, lactose, dulcitol, salicin, glucose, sucrose, galactose, melibiose, maltose, cellobiose, ramnose, salicin, manose, trehalose.^29^^,^^31^ Probiotic strains of *E. faecium* were characterized according to their tolerance to low pH, bile salts and their antibacterial properties.^28^^,^^32^ Confirmation of Entrococcus strain was done by genotypic identification of the strains using RFLP-PCR technique digested with restriction enzyme *Hinf І* (Fig. 1).^33^

**Antimicrobial activity assay. **Before adding the isolated bacteria to food sample, we assayed antimicrobial activity of delivered *E. faecium* by an agar well diffusion test.^35^ *Enterococcus faecium* was grown overnight (18 to 24 hr) in MRS broth at 30 ˚C and the cell free supernatant of bacteria obtained.^34^ Supernatant fluids were adjusted to pH 6.5 by 1 M NaOH (Merck, Darmstadt, Germany). An aliquot of obtained supernatant (100 µL) was loaded into wells cut in BHI (Brain heart infusion; Merck, Darmstadt Germany) agar plates that seeded with the *L. monocytogenes* as indicator strain at a final concentration of 10^5^ CFU per mL.^34^

**UF cheese manufacturing. **Experimental UF cheeses were made in four trials. In each trial four types of cheeses were produced: 1) The control batch (sample numbered 4) using commercial starter culture (1.0 %) and 30 mg kg^-1^ standard bovine rennet (Renco, Eltham, New Zealand), 2) The sample batch (sample numbered 3) containing both probiotic culture of isolated *E. faecium* from Koopeh cheese and the mixture of commercial starter culture (1.0 %) and 30 mg kg^-1 ^rennet, 3) The sample batch (sample numbered 2) containing probiotic culture of isolated *E. faecium* from Koopeh cheese and *L. monocytogenes* (ATCC 19115) in addition mixture of commercial starter culture (1.0 %) and 30 mg kg^-1 ^rennet, 4) The sample batch (sample numbered 1) containing only *L. monocytogenes* without probiotic, in addition starter (1.0 %) and 30 mg kg^-1 ^rennet. The retentate used for production of Iranian UF white cheese was prepared by Iran Dairy Industry Inc., Pegah Company (Urmia, Iran). High microbial quality raw milk was standardized to fat content, and after two steps bactofugation, pasteurization at 72 ˚C for 15 sec and ultra-filtration at 50 ˚C were done. The retentate was pasteurized at 78 ˚C for 15 sec and then cooled to 35 ˚C. Starter culture contained a mixture of mesophilic (G3 mix, composed of *Lactococcus cremoris* and *Lactococcus lactis*) and thermophilic (Yogurt 709, composed of *Streptococcus thermophilus* and *Lactobacillus* *delbrueckii* subsp. *bulgaricus*) cultures (Laboratorium Visby, Tender Aps, Denmark) in the ratio 7:1.^35^

At first, the retentate filled (450 g) in containers, then fresh (18 to 24 hr) *E. faecium* culture was added to the test containers in 10^9 ^CFU per mL equal to McFarland 4 number (12 × 10^8^ CFU per mL). Starter cultures (1.0%) and rennet immediately were added into treatment groups. After that, in order to preventing of line contamination, containers were transferred to microbiology laboratory and 10^5^ CFU per mL of fresh culture (18 to 24 hr) of *L. monocytogenes was *added to the sample containers. All containers were incubated at 30 ˚C for retentate coagulation. After 2 hr, a parchment paper was placed on top of the coagulum of containers and dry salt (3.0 %) was added on it and were sealed with aluminum foil. Cheese packs based on Pegah cheese manufacturing protocol were held at 30 ˚C for 12 hr and inverted for another 12 hr, after that cheese packs were transferred to a cool temperature (4 to 6 ˚C); the second 24 hr was considered as the first day of ripening and the samples were ripened for two months.^35^ One cheese of each trial was sampled at 1, 15, 30, 45 and 60 days during ripening. During the manufacturing process, the initial contamination level of *L. monocytogenes* was ordered 10^5 ^CFU per mL to provide infective dose for human infection.^36^

**Chemical composition. **To evaluate of the effect of added *E. faecium* on chemical composition of UF cheese, pH, moisture, salt, dry matter, fat and protein of samples were measured.^37^^,^^38^

**Statistical analysis. **All analyses were repeated in triplicates. The data were analyzed using Minitab 15 software (Minitab Pty., Sydney, Australia) by the two way analysis variance and GraphPad prism (Version 5.04; GraphPad Inc., San diego, USA). All values were stated as the mean ± SD at the *p* value less than 0.05. 

## Results

**Fig. 2:**
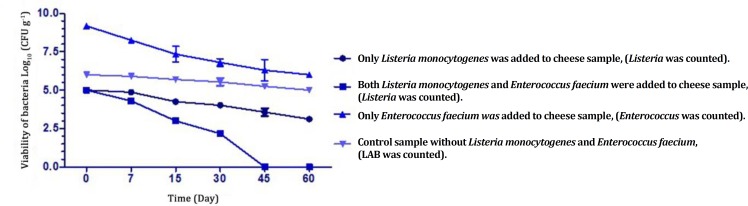
Survival *of L. monocytogenes* and isolated *E. faecium* from Koopeh cheese during ripening period in manufactured UF cheese

*In vitro* antibacterial assay of *E. faecium* showed inhibitory effects on *L. monocytogenes* with obvious growth inhibition zone on BHI agar. Lactic acid bacteria and *L. monocytogenes* counts for all treatment groups are showed in Figure 2 and Table 1. The initial number of LAB in the control sample on MRS and M17 agar was 6 Log g^-1^. The counts started to decrease slightly after day seven (*p* < 0.05) and dropped to a final population of 5 Log g^-1^ at the end of 60 days of storage. After the first day of the cheese production, *E. faecium* was present at 9 Log g^-1^ and remained relatively constant during ripening, and decreased to about 6 Log g^-1^ at the end of 60 days of ripening. On the other hand, the number of *E. faecium *in control cheeses was 6 Log g^-1 ^on day 60. The count of LAB in the test group (mixture of probiotic and starter culture) from 15 Log g^-1 ^of total count reduced to 11 Log g^-1^ at the end of 60 days of ripening. The count of *L. monocytogenes *after 60 days of ripening in blank sample without probiotic strain of *E. faecium* decreased 1 Log but in test samples with protective strain decreased 3 Log in 30 days and reached to zero at 45 days. There were not significant changes in LAB count in all treatment groups (*p < 0.*05), (Table 1). Table 2 shows the results of cheese gross composition at the days 0, 7, 15, 30, 45 and 60 in the ripening period. There were not significant (*p *< 0.05) changes in chemical parameters such as fat, protein and total solid (TS) of UF cheese treatment groups. Slightly increasing at the level of pH from 4.8 at the day zero to 6.6 at the end 60 days of ripening was the common change in enriched cheese with *E. faecium*. Salt percentage increased from 2.7 to 3.3 % in both treatment groups. The moisture content was reduced after seven days from 65 to 63.5 % and remained constant up to 60 days of ripening.

**Table 1:** Viability *of L. monocytogenes* and isolated *E. faecium* from Koopeh cheese during ripening period in ultra-filtrated cheese (Log g^-1^).

**Days**	**Batch number** *								
	**1**	**1-1**	**2**	**2-2**	**2-3**	**3**	**3-3**	**4**	
**0**	5.00 ± 0.00	6.00 ± 0.06	5.00 ± 0.00	9.40 ± 0.10	6.10 ± 0.00	9.18 ± 0.05	6.10 ± 0.16		6.00 ± 0.00
**7**	4.87 ± 0.00	5.80 ± 0.10	4.30 ± 0.05	8.50 ± 0.50	5.90 ± 0.17	8.25 ± 0.10	5.80 ± 0.37		5.90 ± 0.04
**15**	4.25 ± 0.10	5.60 ± 0.35	3.00 ± 0.14	8.00 ± 0.65	5.70 ± 0.45	7.35 ± 0.50	5.70 ± 0.15		5.70 ± 0.15
**30**	4.02 ± 0.05	5.50 ± 0.05	2.18 ± 0.11	7.45 ± 0.45	5.30 ± 0.65	6.80 ± 0.25	5.40 ± 0.15		5.54 ± 0.25
**45**	3.57 ± 0.25	5.20 ± 0.55	-	6.90 ± 0.65	5.10 ± 0.45	6.30 ± 0.70	5.10 ± 0.65		5.25 ± 0.05
**60**	3.12 ± 0.12	5.10 ± 0.07	-	6.40 ± 0.22	4.85 ± 0.28	6.00 ± 0.01	5.00 ± 0.17		5.00 ± 0.00

* 1: Only *L. monocytogenes* was added to cheese sample (*Listeria* was counted); 1-1^:^ Only *L. monocytogenes* was added to cheese sample (LAB was counted); 2: Both *L. monocytogenes* and *E. faecium* were added to cheese sample (*Listeria* was counted); 2-2: Both *L. monocytogenes* and *E. faecium* were added to cheese sample *(E. faecium* was counted); 2-3: Both *L. monocytogenes* and *E. faecium* were added to cheese sample (LAB was counted); 3: Only *E. faecium* was added to cheese sample (*E. faecium* was counted); 3-3: Only *E. faecium* was added to cheese sample (LAB was counted); 4: Control sample without of *L. monocytogenes* and *E. faecium* (LAB was counted).

**Table 2:** Chemical parameters of ultra-filtrated cheese during ripening period

**Retentate**	**Sample No. 2 (** **without** *** E. faecium)***	**Sample No. 1 (** **enriched with ** ***E. faecium)***	**Day**	**Chemical test**
6.5 - - - - -	4.68 ± 0.08 4.77 ± 0.10 6.86 ± 0.06 6.63 ± 0.07 6.26 ± 0.07 6.20 ± 0.10	4.82 ± 0.06 4.76 ± 0.02 6.70 ± 0.03 6.65 ± 0.03 6.57 ± 0.06 6.60 ± 0.10	0 7 15 30 45 60	**pH**
33.73 - - - - -	35.18 ± 0.20 36.01 ± 0.10 36.48 ± 0.32 36.54 ± 0.13 36.70 ± 0.25 36.72 ± 0.30	35.22 ± 0.15 36.28 ± 0.28 36.84 ± 0.27 37.00 ± 0.27 36.78 ± 0.17 37.15 ± 0.10	0 7 15 30 45 60	**Total solid (%)**
41.97 - - - - -	42.00 ± 0.15 42.00 ± 0.15 41.11 ± 0.80 41.05 ± 0.80 40.87 ± 0.55 41.00 ± 0.30	42.00 ± 0.19 42.00 ± 0.20 40.71 ± 0.60 40.54 ± 0.30 40.78 ± 0.25 41.05 ± 0.20	0 7 15 30 45 60	**Fat (%)**
12.69 - - - - -	12.50 ± 0.05 12.52 ± 0.07 12.63 ± 0.05 12.53 ± 0.07 12.39 ± 0.09 12.45 ± 0.05	12.48 ± 0.10 12.62 ± 0.06 12.63 ± 0.05 12.49 ± 0.10 12.78 ± 0.01 12.65 ± 0.01	0 7 15 30 45 60	**Protein (%)**
- - - - - -	2.60 ± 0.06 3.20 ± 0.10 3.25 ± 0.10 3.20 ± 0.10 3.26 ± 0.15 3.24 ± 0.10	2.70 ± 0.15 3.30 ± 0.11 3.27 ± 0.15 3.23 ± 0.10 3.28 ± 0.10 3.28 ± 0.10	0 7 15 30 45 60	**Salt (%)**
66.27 - - - - -	64.80 ± 0.05 63.99 ± 0.26 63.52 ± 0.25 63.46 ± 0.26 63.30 ± 0.11 63.35 ± 0.10	65.00 ± 0.02 63.72 ± 0.72 63.16 ± 0.33 63.00 ± 0.43 63.22 ± 0.22 63.35 ± 0.21	0 7 15 30 45 60	**Moisture (%)**

## Discussion

Screening of traditional foods is generally an effective tool for the identification of useful bacteria strains for industrial and scientific purposes.^39^ In recent years, special attention has been made to improve the quality and quantity of dairy products, including functional foods that contains probiotics and have beneficial effects on consumer’s health. 

The UF cheese recognized as a highly consumed industrial cheese and an important dairy product in Iran. Enrichment of this type of cheese with useful bacteria such as probiotics with ability of surviving and improving cheese quality and its physicochemical properties helps to enhance the cheese popularity among consumers. Usually enrichment of cheeses is implemented by commercial strains of probiotics or adjunct cultures. In present study enrichment of UF cheese was done by isolated *E. faecium* as a native lactic acid bacteria derived from traditional Koopeh cheese. 

Changes in count of *E. faecium* were shown in Table 1. In ripening period of UF cheese the number of *Enterococcus* declined (*p *< 0.05). Results presented in Table 1 reveal that there has been statistically significant difference between first 30 days the end days of ripening, in the presence of protective culture of *E. faecium* in manufactured UF cheese. Probably the main reason of reducing in *Enterococcus* count was the high salt content and low pH in cheese causing growth inhibition of unprotected added probiotics in UF cheese.^40^ The results was in agreement with the results of other scientists.^17^^,^^20^^,^^41^ Ozer *et al*. in 2009 reported 3 Log decreasing of unprotected probiotics and 1 Log decreasing of encapsulated probiotics in Turkish white cheese within 90 days of storage period.^41^ During the first 15 days of storage period, the count of *E. faecium* decreased rapidly. After that because of their adaptation to the conditions, the count change was lesser. The results indicated that added *E. faecium* did not exert any effect on the growth of commercial starters (Table 1).

This study indicates an obvious antagonistic activity towards *L. monocytogenes* by *E. faecium*. Existence of inhibition zone on BHI agar demonstrated inhibitory extracellular metabolites synthesized by *E. faecium*. Most* Enterococci *from dairy products have also been reported having antimicrobial activity against a broad spectrum of spoilage and pathogenic organisms such as *L. monocytogenes, S. aureus, Clostridium *spp., and *Bacillus *spp.^42^^-^^46^ Specific enterococci strains have been used as probiotic adjunct cultures in Cheddar cheese because of their ability to improve the intestine microbial balance.^10^^,^^11^^, ^^47^^,^^48^

Chemical composition and changes of the cheeses during ripening has been shown in Table 2. The chemical analysis of two cheese groups in this study showed that the addition of the tested probiotic microorganisms had no unfavorable effect on cheese composition. Similar results were reported in previous studies implemented by Zomorodi *et al*., Kasimoglu *et al.* and Ong *et al*.^6^^,^^17^^,^^28^ The moisture decreased and salt percentage increased significantly in all samples during ripening period (*p *< 0.05), due to osmotic flow of salt water during cheeses storage and transferring salt in and water to out of the cheese texture. There was no significant difference from a texture and flavor point of view between the control and experimental cheeses. pH increased slightly due to proteolytic activity of *E. faecium*.^6^ Protein content did not change during ripening period. The ratio of fat to dry matter in cheese samples due to lipolysis and transferring of fatty acids of cheese to salt water decreased slightly.^28^

In conclusion, the results showed that *E. faecium* of Koopeh cheese is suitable for development of an acceptable probiotic UF cheese and can be adapted to industrial production of UF cheese. The final count of *E. faecium* after 60 days storage of UF cheese samples was 10^6 ^CFU per g confirming its viability. Enrichment caused by *E. faecium* in some chemical parameters of manufactured UF cheese was not significant. 
